# Characterization of HAF-4- and HAF-9-localizing organelles as distinct organelles in *Caenorhabditis elegans* intestinal cells

**DOI:** 10.1186/s12860-015-0076-2

**Published:** 2016-01-27

**Authors:** Takahiro Tanji, Kenji Nishikori, Syoko Haga, Yuki Kanno, Yusuke Kobayashi, Mai Takaya, Keiko Gengyo-Ando, Shohei Mitani, Hirohisa Shiraishi, Ayako Ohashi-Kobayashi

**Affiliations:** Department of Immunobiology, School of Pharmacy, Iwate Medical University, 2-1-1 Nishi-tokuta, Yahaba, Shiwa-gun, Iwate 028-3694 Japan; Department of Physiology, School of Medicine, Tokyo Women’s Medical University, 8-1 Kawada-cho, Shinjuku-ku, Tokyo, 162-8666 Japan; Present address: Saitama University Brain Science Institute, 255 Shimo-okubo, Sakura-ku, Saitama, 338-8570 Japan

**Keywords:** ABC transporter, *Caenorhabditis elegans*, Intestine, Lipid droplet, Lysosome, Yolk protein, Endosome, Rab7

## Abstract

**Background:**

The intestinal cells of *Caenorhabditis elegans* are filled with heterogeneous granular organelles that are associated with specific organ functions. The best studied of these organelles are lipid droplets and acidified gut granules associated with GLO-1, a homolog of the small GTPase Rab38. In this study, we characterized a subset of the intestinal granules in which HAF-4 and HAF-9 localize on the membrane. HAF-4 and HAF-9 are ATP-binding cassette (ABC) transporter proteins that are homologous to the mammalian lysosomal peptide transporter TAPL (transporter associated with antigen processing-like, ABCB9).

**Results:**

Using transgenic worms expressing fluorescent protein-tagged marker proteins, we demonstrated that the HAF-4- and HAF-9-localizing organelles are not lipid droplets and do not participate in yolk protein transport. They were also ruled out as GLO-1-positive acidified gut granules. Furthermore, we clarified that the late endosomal protein RAB-7 localizes to the HAF-4- and HAF-9-localizing organelles and is required for their biogenesis.

**Conclusions:**

Our results indicate that the HAF-4- and HAF-9-localizing organelles are distinct intestinal organelles associated with the endocytic pathway.

**Electronic supplementary material:**

The online version of this article (doi:10.1186/s12860-015-0076-2) contains supplementary material, which is available to authorized users.

## Background

The intestine of *Caenorhabditis elegans* is a multifunctional organ involved in the uptake, metabolism, storage, and distribution of nutrients, as well as in detoxification and immune defense [1]. It consists of a tube comprising only 20 epithelial cells that are not replaced after embryonic development; thus, each intestinal cell is considered to have multiple functions throughout the life of the animal. The intestinal cells are filled with heterogeneous granular organelles that are associated with specific organ functions [2]. Therefore, to understand how these individual cells achieve multiple intestinal functions, these granular organelles should be classified and their physiological roles determined. However, the relationships between different types of granules, their physiological roles, and the regulation of their biogenesis are largely unknown.

The best studied of the intestinal granular organelles are the acidified organelles known as “gut granules”, which are easily recognized by the presence of autofluorescent materials [3, 4]. Gut granules are regarded as lysosome-related organelles, because GLO-1 localizes to embryonic gut granules, and is required for the biogenesis of the organelles. GLO-1 is a member of the RAB family of small G proteins and a homolog of mammalian Rab38; it is required for the biogenesis of melanosomes (lysosome-related organelles in melanocytes). Several other genetic factors are required for gut granule biogenesis [5–9]. Although some of the physiological roles of gut granules have been identified, such as the storage of zinc [10] and cholesterol [11], and signaling for aging [12], their overall functions remain poorly understood.

Other well-studied intestinal granules are the fat storage organelles known as lipid droplets [13]. The intestine is the major organ responsible for fat storage. Similar to mammalian lipid droplets, those of *C. elegans* are characterized by a phospholipid monolayer membrane [14]. The short-chain dehydrogenase DHS-3 and the triglyceride lipase ATGL-1 were recently identified as marker proteins that localize on the membrane of lipid droplets [15, 16].

Previously, we reported that HAF-4 and HAF-9, ATP-binding cassette (ABC) transporter proteins that are homologous to the mammalian lysosomal peptide transporter TAPL (transporter associated with antigen processing like, ABCB9), localize to the membrane of a subset of intestinal granular organelles approximately 2 μm in diameter from the late larval to adult stages [17]. HAF-4 and HAF-9 are involved in maintaining the normal formation of their localizing organelles, probably by forming a heterodimer as a functional unit of a transporter [17, 18]. The colocalization of HAF-4, HAF-9, and LMP-1, a homolog of mammalian lysosome-associated membrane proteins (LAMPs), suggests their relevance to lysosomes. This interpretation is further supported by the observation that HAF-4 and HAF-9 localize to the enlarged vacuole formed in the mutant of *ppk-3*, a gene regulating the terminal maturation of lysosomes. Furthermore, HAF-4- and HAF-9-localizing organelles are neither acidified nor autofluorescent granules, and the mutants of *haf-4* and *haf-9* show the same or a greater number of acidified and autofluorescent granules as the wild-type, suggesting that the HAF-4- and HAF-9-localizing organelles are distinct from gut granules. We also reported that the HAF-4- and HAF-9-localizing organelles did not show positive vital staining for the lysochrome dye Nile Red; however, a subsequent study demonstrated that fixed Nile Red staining, but not vital staining, showed specificity to the lipid droplets in the cells [13, 14]. Therefore, the possibility that HAF-4- and HAF-9-localizing organelles are lipid droplets remained an open question.

Here, we report the classification of the HAF-4- and HAF-9-localizing organelles among intestinal granules using transgenic worms expressing fluorescent protein-tagged marker proteins. We confirmed that these organelles are not lipid droplets and do not participate in yolk protein transport. They are also not GLO-1-positive gut granules. Further investigation using the late endosomal protein RAB-7 revealed that the HAF-4- and HAF-9-localizing organelles are distinct intestinal organelles associated with the endocytic pathway.

## Results

### HAF-4- and HAF-9-localizing organelles are not lipid droplets

**Fig. 1:**
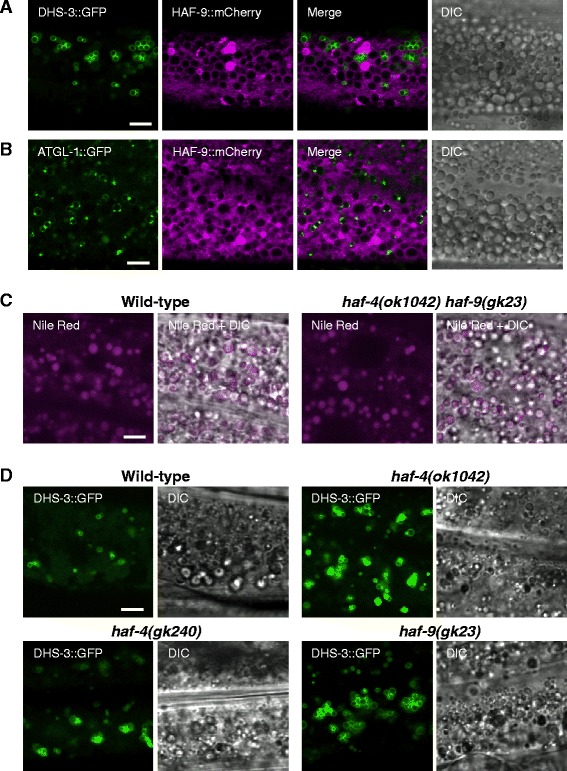
HAF-4- and HAF-9-localizing intestinal organelles are distinct from lipid droplets. **a** and **b** Comparison of the localization of HAF-9::mCherry with the lipid droplet markers DHS-3::GFP (**a**) and ATGL-1::GFP (**b**) in *Is[dhs-3::GFP]/Is[haf-9::mCherry]* and *Is[atgl-1::GFP];Is[haf-9::mCherry]*, respectively. DHS-3::GFP or ATGL-1::GFP (green), HAF-9::mCherry (magenta), the merged images, and the corresponding DIC images are presented. DHS-3::GFP and HAF-9::mCherry did not localize to the surface of identical intestinal granules. **c** Nile Red staining of the wild-type N2 (left) and the *haf-4(ok1042) haf-9(gk23)* double-mutant worms (right). Nile Red (magenta) and the merged images with DIC are presented. Nile Red-positive intestinal granules were intact even in the absence of the HAF-4- and HAF-9-localizing organelles. **d** Expression of the lipid droplet marker DHS-3::GFP in the wild-type (top left), *haf-4(ok1042)* (top right), *haf-4(gk240)* (bottom left), and *haf-9(gk23)* (bottom right) worms. DHS-3::GFP-positive lipid droplets did not decrease in these mutants. Bars, 5 μm

We determined whether the HAF-4- or HAF-9-localizing organelles are lipid droplets by Nile Red staining of HAF-4::green fluorescent protein (GFP)- and HAF-9::GFP-expressing transgenic worms following fixation of the worms in paraformaldehyde. Neither HAF-4-positive nor HAF-9-positive intestinal granules were stained with Nile Red (Additional file 1: Figure S1). We also compared the localization of HAF-9::mCherry with that of the lipid droplet markers DHS-3::GFP [15] and ATGL-1::GFP [16]. DHS-3::GFP did not localize to the surface of either the HAF-9::mCherry-positive granules (Fig. 1a) or the autofluorescent granules, which are regarded as acidified gut granules (Additional file 1: Figure S2). Furthermore, HAF-9::mCherry also did not localize to the surface of the ATGL-1::GFP-positive granules (Fig. 1b). These results indicate that the HAF-4- and HAF-9-localizing organelles are not lipid droplets.

We next determined whether HAF-4 and HAF-9 are involved in the biogenesis of lipid droplets using deletion mutants of *haf-4* and *haf-9*, which exhibit defects in the biogenesis of the HAF-4- and HAF-9-localizing organelles [17]. These mutants did not show a decrease in the number of lipid droplets, as shown by Nile Red staining (Fig. 1c) and DHS-3::GFP localization (Fig. 1d), indicating that the organelles are not required for the biogenesis of lipid droplets. This conclusion was also supported by the normal formation of DHS-3::GFP-positive lipid droplets in the *lmp-1* mutant, in which HAF-4- and HAF-9-localizing organelles show morphological aberrations [17] (Additional file 1: Figure S3).

### HAF-4- and HAF-9-localizing organelles are irrelevant to yolk protein transport

**Fig. 2:**
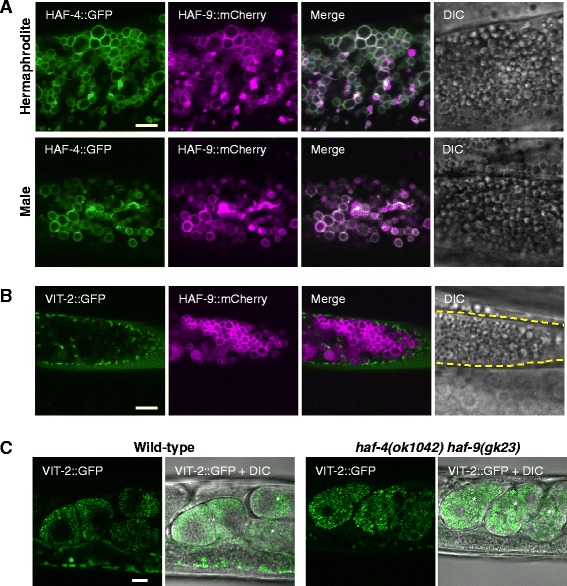
HAF-4- and HAF-9-localizing intestinal organelles are irrelevant to the storage and distribution of yolk granules. **a** Localization of HAF-4::GFP (green) and HAF-9::mCherry (magenta) to the intestinal granules in the day-1 adult hermaphrodite (top) and male (bottom) worms of *Is[haf-4::GFP];Is[haf-9::mCherry]*. The merged images and the corresponding DIC images are also presented. HAF-4::GFP and HAF-9::mCherry colocalized on the surface of intestinal granules not only in the hermaphrodites but also in the males. Bar, 5 μm. **b** Comparison of the localization of HAF-9::mCherry with VIT-2::GFP in *Is[vit-2::GFP];Is[haf-9::mCherry]*. VIT-2::GFP (green), HAF-9::mCherry (magenta), the merged image, and the corresponding DIC image are presented. VIT-2::GFP did not localize to the HAF-9::mCherry-positive intestinal granules. Dashed lines indicate the boundary of the intestine. Bar, 5 μm. **c** The localization of VIT-2::GFP in the wild-type (left) and *haf-4(ok1042)haf-9(gk23)* (right) worms. VIT-2::GFP and the merged images with corresponding DIC images are presented. Developing embryos positive for VIT-2::GFP are indicated by asterisks. Distribution of VIT-2::GFP to the embryos was unaltered even in the mutant worms. Bar, 10 μm

A previous report indicated that deletion mutants of *haf-4* and *haf-9* show a reduced brood size [17], which suggests the involvement of the HAF-4- and HAF-9-localizing organelles in the nutrition supply to oocytes. However, these organelles are found in both hermaphrodites and males (Fig. 2a and Additional file 1: Figure S13a). In *C. elegans*, VIT-2 (a yp170B homolog) is secreted from the intestinal cells into the pseudocoelomic space, and is taken up by developing oocytes through gonadal sheath cells as a nutritional source [19]. VIT-2 is expressed only in the intestinal cells of the adult hermaphrodite. Although the mechanism by which the oocytes uptake yolk protein via receptor-mediated endocytosis is well characterized [20], precisely how the yolk protein is pooled and trafficked in the intestinal cells has not been elucidated in detail. The yolk protein in the intestinal cells was visualized by VIT-2::GFP, which was distinguishable from the autofluorescent granules and lipid droplets (Additional file 1: Figure S4). However, VIT-2::GFP did not localize to either the HAF-9::mCherry-positive (Fig. 2b and Additional file 1: Figure S13b) or LMP-1::mRFP-positive granules (Additional file 1: Figure S5). Furthermore, the uptake of VIT-2::GFP in embryos was unaffected in the *haf-4 haf-9* double mutant (Fig. 2c). These results suggest that the HAF-4- and HAF-9-localizing organelles are irrelevant to yolk protein transport.

### HAF-4- and HAF-9-localizing organelles are not acidified gut granules

**Fig. 3:**
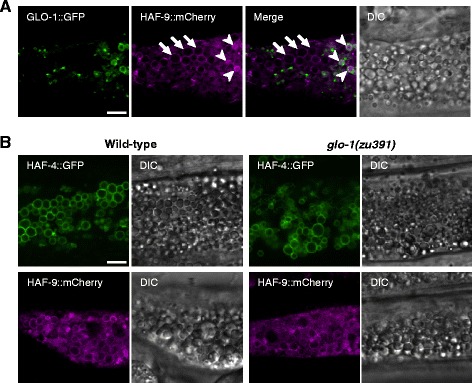
HAF-4- and HAF-9-localizing intestinal organelles are not acidified gut granules. **a** Comparison of the localization of HAF-9::mCherry with GLO-1::GFP in *Is[ges-1p::glo-1::GFP];Is[haf-9::mCherry]*. GLO-1::GFP (green), HAF-9::mCherry (magenta), the merged image, and the corresponding DIC image are presented. GLO-1::GFP did not localize to the mCherry-edged intestinal granules (arrows). Arrowheads indicate the accumulation of mCherry in the gut granules. **b** The localization of HAF-4::GFP (top) and HAF-9::mCherry (bottom) in the wild-type (left) and *glo-1(zu391)* (right) worms. The corresponding DIC images are also presented. HAF-4::GFP-positive and HAF-9::mCherry-positive intestinal granules were observed even in the mutant worms. Bars, 5 μm

Although the HAF-4- and HAF-9-localizing organelles are not acidified, LMP-1, a homolog of mammalian LAMPs, also localizes to these organelles, suggesting a potential relationship with the lysosomes [17]. HAF-4 and HAF-9 do not localize to organelles with autofluorescent materials that are characteristic of gut granules, and *haf-4* and *haf-9* were found to be unnecessary for the formation of gut granules. To investigate the relationship between these organelles and gut granules in more detail, we compared the localization of HAF-4 and HAF-9 with that of GLO-1, a small GTPase required for the biogenesis of gut granules. GLO-1::GFP localized to the surface of autofluorescent granules but not to the Nile Red-positive lipid droplets (Additional file 1: Figure S6). In adult worms expressing both GLO-1::GFP and HAF-9::mCherry, the mCherry-edged intestinal granules (arrows in Fig. 3a) and the mCherry-internalized intestinal granules (arrowheads in Fig. 3a) were observed, the latter of which are thought to be lysosomes or lysosome-related organelles accumulating mCherry in their lumen. Although GLO-1::GFP localized to the surface of the mCherry-internalized granules, it did not localize to the mCherry-edged intestinal granules where HAF-9::mCherry was localized on the membrane (Fig. 3a and Additional file 1: Figure S13c). Similarly, GLO-1::GFP did not localize to the LMP-1::mRFP-edged intestinal granules (Additional file 1: Figure S7). We also investigated the localization of HAF-4 and HAF-9 in the *glo-1* mutant. The localization of HAF-4::GFP and HAF-9::mCherry to the surface of intestinal granules was observed even in the *glo-1(zu391)* mutants (Fig. 3b and Additional file 1: Figure S13d-e), as was the localization of DHS-3::GFP (Additional file 1: Figure S8). These results indicate that the HAF-4- and HAF-9-localizing organelles are not acidified gut granules.

### HAF-4- and HAF-9-localizing organelles are associated with the endocytic pathway

Although the HAF-4- and HAF-9-localizing organelles are prominent intestinal granules in late larval and young adult worms, they are distinct from acidified gut granules and lipid droplets*.* Furthermore, HAF-4::GFP and HAF-9::mCherry were not localized to either the peroxisome marker-positive organelles or the mitochondrion marker-positive organelles (Additional file 1: Figure S9). Therefore, what are these organelles?

**Fig. 4:**
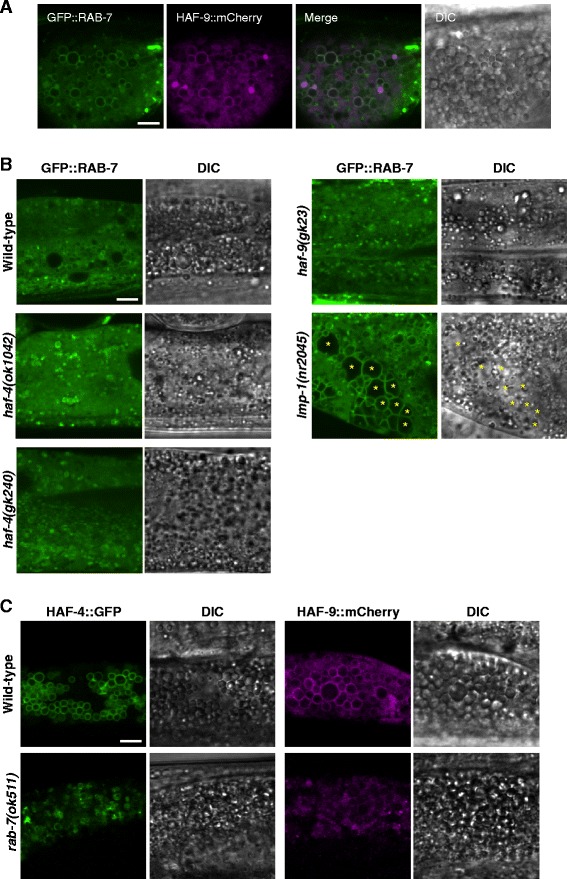
HAF-4- and HAF-9-localizing organelles are associated with the endocytic pathway. **a** Co-localization of GFP::RAB-7 and HAF-9::mCherry in *Is[haf-9::mCherry];Ex[GFP::rab-7]*. GFP::RAB-7 (green) HAF-9::mCherry (magenta), the merged image, and the corresponding DIC image are presented. **b** Localization of GFP::RAB-7 in the wild-type (top left) *haf-4(ok1042)* (middle left), *haf-4(gk240)* (bottom left), *haf-9(gk23)* (top right), and *lmp-1(nr2045)* (middle right). GFP::RAB-7 (green) and the corresponding DIC images are presented. In the *haf-4* and *haf-9* mutants, exhibiting defects in the HAF-4- and HAF-9-localizing organelles, the number of intestinal granules with GFP fluorescence on their surface decreased. In *lmp-1(nr2045)*, GFP fluorescence was detected on the surface of the enlarged vacuole (asterisks). **c** Subcellular localization of HAF-4::GFP (left) and HAF-9::mCherry (right) in the wild-type (top) and *rab-7(ok511)* (bottom). The corresponding DIC images are also presented. HAF-4::GFP-positive and HAF-9::mCherry-positive intestinal granules dramatically decreased in the *rab-7* mutant. Bars, 5 μm

The localization of HAF-4 and HAF-9 to the enlarged vacuoles in *ppk-3(n2668)* mutants suggests their involvement in the endocytic pathway [17]. PPK-3 regulates terminal lysosome maturation and the *ppk-3* mutation results in the enlargement of vacuoles where LMP-1 and RAB-7 localize [21]. We compared the localization of HAF-9::mCherry with that of the late-endosomal and lysosomal marker GFP::RAB-7, which did not localize to autofluorescent granules or Nile Red-positive lipid droplets (Additional file 1: Figure S10). Although the GFP::RAB-7 signal showed a more dispersed pattern than that of HAF-9::mCherry, GFP::RAB-7 and HAF-9::mCherry colocalized to the surface of intestinal granules (Fig. 4a and Additional file 1: Figure S13f). Both the altered localization of GFP::RAB-7 in the *haf-4* and *haf-9* mutants and the localization of GFP::RAB-7 to the enlarged organelles in the *lmp-1(nr2045)* mutant strongly support the localization of GFP::RAB-7 to the HAF-4- and HAF-9-localizing organelles. The localization of HAF-4::GFP and HAF-9::mCherry to the surface of intestinal granules was severely impaired in the *rab-7* mutant (Fig. 4c and Additional file 1: Figure S13g-h), as was that of LMP-1::mRFP (Additional file 1: Figure S11). In contrast, the autofluorescent granules and DHS-3::GFP-positive lipid droplets were not affected in the *rab-7* mutant (Additional file 1: Figure S12).

## Discussion

We investigated the relationships among the intestinal granular organelles of *C. elegans* using two approaches. The first was through comparison of the localization of fluorescent protein-tagged proteins and fluorescently visualizable materials inside the organelles, facilitating the identification of organelles to clarify the interrelationships in their formation processes. The other approach involved investigating how a defect in a subset of intestinal organelles affected other subsets, which could help to determine whether or not these organelles require each other for their biogenesis and function.

These investigations clearly showed that the HAF-4- and HAF-9-localizing organelles are not lipid droplets, based on the lack of Nile Red stainability and DHS-3::GFP localization, and that they are not required for the biogenesis and lipid storage of lipid droplets. It was also confirmed that the organelles are not yolk granules and are not necessary for the transport of yolk protein to developing oocytes. The involvement of these organelles in the storage or distribution of nutrients has been suggested, because *haf-4* and *haf-9* mutants, which are defective in organelle biogenesis, produced phenotypes with slow growth and reduced brood size. However, the organelles seem to be irrelevant for the storage and distribution of lipids and yolk protein. The storage of polypeptides and/or carbohydrates is a possible candidate for their function. In particular, the storage of peptides seems to be a plausible function, because HAF-4 and HAF-9 are believed to transport peptides from the cytosol to the lumen of the organelles, based on their homology with the mammalian peptide transporter TAPL [17].

Furthermore, HAF-4- and HAF-9-positive intestinal granules did not overlap with GLO-1-positive granules, and the HAF-4- and HAF-9-localizing organelles and acidified gut granules did not require each other for their biogenesis, suggesting that they are distinct organelles that are formed by different pathways. Experiments using GFP::RAB-7 and the *rab-7* mutants demonstrated that RAB-7 associates with HAF-4- and HAF-9-localizing organelles and participates in their biogenesis. The small GTPase RAB-7 is a key regulator of the endocytic pathway, and replacement of RAB-5 with RAB-7 occurs when early endosomes mature into late endosomes [22]. Therefore, our results strongly suggest that the HAF-4- and HAF-9-localizing organelles are formed through the maturation or fusion of RAB-7-positive late endosomes. Transport substrates of HAF-4 and HAF-9 may possibly be necessary for the maturation or fusion process. The multivesicular structure observed in the *lmp-1* mutant under a transmission electron microscope also suggested a defect in the endocytic pathway [17]. More interestingly, the biogenesis of acidified gut granules requires the Rab38 homologue GLO-1 but not RAB-7 [9], providing further evidence that HAF-4- and HAF-9-localizing organelles and acidified gut granules are irrelevant. Mammalian Rab38 associates with melanosomes and platelet dense granules and is necessary for their biogenesis [23, 24]. In contrast to the acidified gut granules, which are lysosome-related organelles, HAF-4- and HAF-9-localizing organelles are thought to be more related to the canonical lysosomes in terms of their biogenesis, even though they are not acidified and are much larger than the average lysosome (approximately 2 μm versus 0.05–0.5 μm in diameter). The possibility that they are residual bodies is also unlikely because autofluorescent materials known as age pigments, which are characteristic of residual bodies [25], are attributed to acidified gut granules but not to HAF-4- and HAF-9-localizing organelles.

## Conclusions

Although the physiological function of the HAF-4- and HAF-9-localizing organelles remains elusive despite their abundance in intestinal cells, our data demonstrate that they are distinct intestinal organelles associated with the endocytic pathway. The storage of polypeptides and/or carbohydrates is a possible candidate for their function. Uncovering the specific function of the organelles and their relationship with other intracellular compartments would provide insight into the organelle diversity to support the multifunctionality of *C. elegans* intestinal cells.

## Methods

### General methods and establishment of mutant and transgenic strains

The maintenance, husbandry, and genetic crosses of *C. elegans* were performed according to the standard protocols described by Brenner [26]. Strains were cultured at 20 °C unless otherwise noted. Bristol strain N2 was used as the standard wild-type strain, and the following mutant and transgenic strains were obtained from the *Caenorhabditis* Genetics Center (University of Minnesota): *haf-4(ok1042)*, *haf-4(gk240)*, *haf-9(gk23)*, *lmp-1(nr2045)*, *glo-1(zu391)*, *rab-7(ok511)*, *hjIs67[atgl-1::GFP + mec-7::mRFP]*, *bIs1[vit-2::GFP + rol-6(su1006)]*, *hjIs9[ges-1p::glo-1::GFP + unc-119(+)]*, *hjIs37[vha-6p::mRFP-PTS1 + Cbr-unc-119(+)]*, *hjIs8[ges-1p::GFP-PTS1]*, and *zcIs17[ges-1::GFP(mit)]*. The transgenic strains *Is[haf-4::GFP + rol-6(su1006)]*, *Is[haf-9::GFP + rol-6(su1006)]*, *Is[haf-9::mCherry + rol-6(su1006)]*, and *Is[ges-1p::lmp-1::mRFP + rol-6(su1006)]* were established as described by Kawai et al. [17] and Tanji et al. [18]. *Is[dhs-3::GFP]* was provided by Dr. Liu (Institute of Biophysics, Chinese Academy of Sciences, Beijing, China).

To generate the *GFP::rab-7* construct in which enhanced green fluorescent protein (EGFP) is fused in-frame at the N-terminus of RAB-7, three DNA fragments were polymerase chain reaction (PCR)-amplified and fused as follows. A 1.6-kb DNA fragment (corresponding to the promoter region of *rab-7*) and a 1.9-kb DNA fragment (corresponding to the coding sequence and 3′ untranslated region of *rab-7*) were PCR-amplified with the primer sets rab-7_A#F3/rab-7_A#R3 and rab-7_A#F1/rab-7_A#R1, respectively. A 0.7-kb sequence of the EGFP coding region was PCR-amplified with the primer set rab-7_A#F2/ rab-7_A#R2. These DNA fragments were fused by PCR sewing with the primer set rab-7_A#F3/ rab-7_A#R1, and subsequently cloned between the *Not*I and *Bam*HI sites of the pBlueScript vector.

The primer sequences used for establishment of the constructs were as follows:

**Table:** 

rab-7_A#F1	gcatggacgagctgtacaagATGTCGGGAACCAGAAAGAAG
rab-7_A#R1	gcagcccgggggatccTTCCAGACGCCAATTGAGAG
rab-7_A#F2	AAAAGGCTTCCAGTGAACAAAAatggtgagcaagggcgagga
rab-7_A#R2	CTTCTTTCTGGTTCCCGACATcttgtacagctcgtccatgc
rab-7_A#F3	accgcggtggcggccgcATTCGCGCCATTTACCTCAAA
rab-7_A#R3	tcctcgcccttgctcaccatTTTTGTTCACTGGAAGCCTTTT

Microinjection of DNA into the *C. elegans* germ line was performed as described by Mello et al. [27] using pRF4 [*rol-6(su1006)*] as a selection marker. Integrant formation was performed according to Mitani’s method [28]. Our study does not require any animal ethics approval.

### Nile Red staining

The worms were washed with S-basal (50 mM potassium phosphate buffer [pH 6.0] containing 0.1 M NaCl) and fixed with 0.5 % paraformaldehyde in phosphate-buffered saline for 1 h at room temperature. Fixed worms were rinsed with M9 buffer (42 mM Na_2_HPO_4_, 22 mM KH_2_PO_4_, 86 mM NaCl, 1 mM MgSO_4_, 0.02 % gelatin) and stored at 4 °C until required. They were stained with 1 μg/mL Nile Red in M9 buffer prepared from a stock solution (1 mg/mL Nile Red in acetone) for 15–30 min at room temperature.

### Optical microscopic observation

The differential interference contrast (DIC) and autofluorescence images were obtained using an epifluorescence microscope (BX51; Olympus Corp., Tokyo, Japan) with DIC optics. Fluorescence images of GFP, mCherry, mRFP, and Nile Red were obtained using a confocal microscope (FV1000; Olympus Corp., Tokyo) with 473-nm or 559-nm laser excitation. Spectral scanning and unmixing were performed using the spectral deconvolution program in the FV1000 software FV10-ASW. For the unmixing from autofluorescence, autofluorescence was enhanced by pre-exposure to ultraviolet rays. All images were obtained using hermaphrodites on adult day 1 after anesthetization with 50 mM NaN_3_ in M9 buffer, unless otherwise indicated. The *glo-1::GFP* and *GFP::rab-7* transgenic worms subjected to the microscopic investigation were reared at 16 °C for clearer identification. All images presented are of the middle part of the intestine (int3 to int7) unless otherwise noted, oriented with the anterior to the left. The presented images are the representatives of at least three animals.

## Additional file

Additional file 1: Figure S1. Staining of *Is[haf-4::GFP]* (a) and *Is[haf-9::GFP]* (b) with the lysochrome dye Nile Red after fixation. **Figure S2.** DHS-3-positive intestinal granules are not autofluorescent granules. **Figure S3.** *lmp-1* is not required for the biogenesis of DHS-3::GFP-positive lipid droplets. **Figure S4.** VIT-2::GFP did not localize to either the autofluorescent granules or the lipid droplets. **Figure S5.** LMP-1::mRFP did not localize to VIT-2::GFP-positive granules. **Figure S6.** GLO-1::GFP-positive granules are autofluorescent but not Nile Red-positive. **Figure S7.** LMP-1::mRFP did not localize to GLO-1::GFP-positive granules. **Figure S8.** *glo-1* is not required for the biogenesis of DHS-3::GFP-positive lipid droplets. **Figure S9.** HAF-4 and HAF-9 did not localize to either the peroxisomes or the mitochondria. **Figure S10.** GFP::RAB-7 did not localize to either the autofluorescent granules or the Nile Red-positive lipid droplets. **Figure S11.** *rab-7* is required for the biogenesis of LMP-1::mCherry-positive granules. **Figure S12.** *rab-7* is not required for the biogenesis of autofluorescent granules and DHS-3::GFP-positive lipid droplets. **Figure S13.** Other representative images. (PDF 5985 kb)

## Abbreviations

ABC: ATP-binding cassette
DIC: Differential interference contrast
GFP: Green fluorescent protein
LAMP: Lysosome-associated membrane protein
PCR: Polymerase chain reaction
TAPL: Transporter associated with antigen processing-like
