# Supplementary material for: Characterization of HAF-4- and HAF-9-localizing organelles as distinct organelles in Caenorhabditis elegans intestinal cells
Source: BMC Cell Biol. 2016 Jan 27;17:4. doi: 10.1186/s12860-015-0076-2 (PMC4729119; doi:10.1186/s12860-015-0076-2)
Supplement: Additional file 1: Figure S1. — Staining of Is[haf-4::GFP] (a) and Is[haf-9::GFP] (b) with the lysochrome dye Nile Red after fixation. Figure S2. DHS-3-positive intestinal granules are not autofluorescent granules. Figure S3. lmp-1 is not required for the biogenesis of DHS-3::GFP-positive lipid droplets. Figure S4. VIT-2::GFP did not localize to either the autofluorescent granules or the lipid droplets. Figure S5. LMP-1::mRFP did not localize to VIT-2::GFP-positive granules. Figure S6. GLO-1::GFP-positive granules are autofluorescent but not Nile Red-positive. Figure S7. LMP-1::mRFP did not localize to GLO-1::GFP-positive granules. Figure S8. glo-1 is not required for the biogenesis of DHS-3::GFP-positive lipid droplets. Figure S9. HAF-4 and HAF-9 did not localize to either the peroxisomes or the mitochondria. Figure S10. GFP::RAB-7 did not localize to either the autofluorescent granules or the Nile Red-positive lipid droplets. Figure S11. rab-7 is required for the biogenesis of LMP-1::mCherry-positive granules. Figure S12. rab-7 is not required for the biogenesis of autofluorescent granules and DHS-3::GFP-positive lipid droplets. Figure S13. Other representative images. (PDF 5985 kb) [file 12860_2015_76_MOESM1_ESM.pdf]

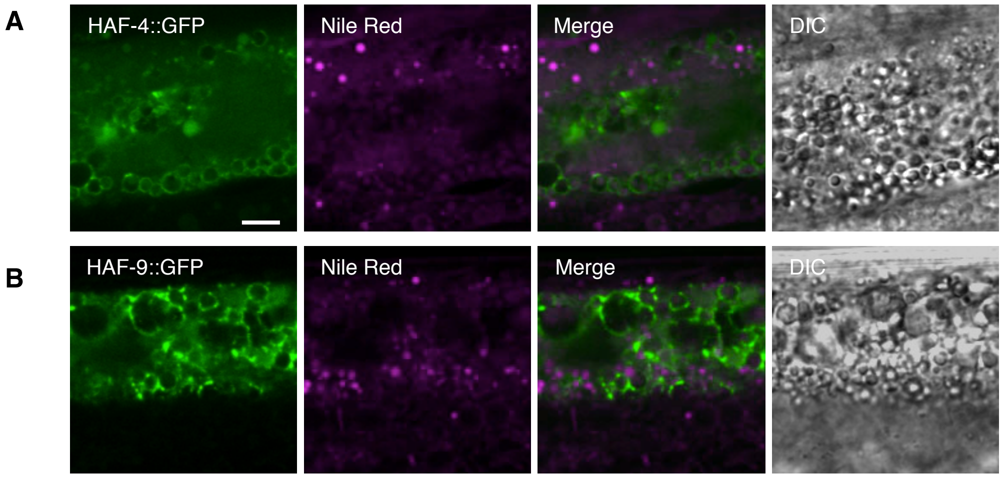

Figure S1 Staining of *Is[haf-4::GFP]* (**a**) and *Is[haf-9::GFP]* (**b**) with the lysochrome dye Nile Red after fixation.
HAF-4::GFP or HAF-9::GFP (green), Nile Red (magenta), the merged images, and corresponding DIC images are presented. Neither HAF-4::GFP-positive nor HAF-9::GFP-positive intestinal granules were stained with Nile Red. Bar, 5 μm.

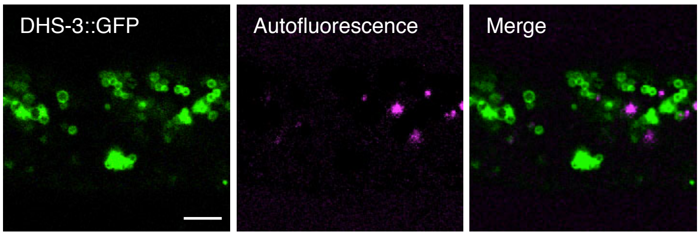

Figure S2 DHS-3::GFP-positive intestinal granules are not autofluorescent granules. Fluorescence observed in the int1 cell of *Is[dhs-3::GFP]* under confocal microscope was unmixed to GFP (green) and autofluorescence (magenta). The merged image is also presented. Bar, 5 μm.

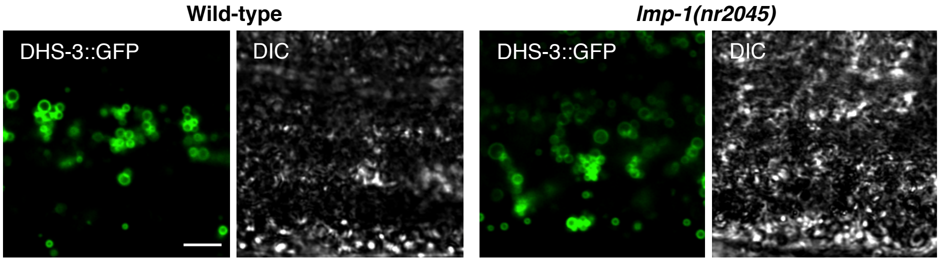

Figure S3 *lmp-1* is not required for the biogenesis of DHS-3::GFP-positive lipid droplets. DHS-3::GFP (green) and the corresponding DIC images in *Is[dhs-3::GFP]* (left) and *Is[dhs-3::GFP];lmp-1(nr2045)* (right) are presented. Bar, 5 μm.

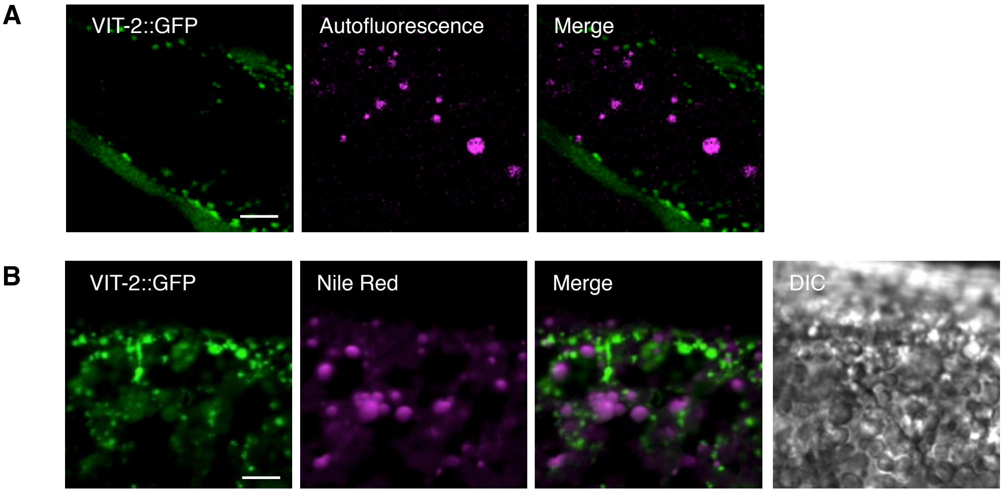

Figure S4 VIT-2::GFP did not localize to either the autofluorescent granules or the lipid droplets. (**a**) Fluorescence observed in *Is[vit-2::GFP]* under confocal microscope was unmixed to GFP (green) and autofluorescence (magenta). The merged image is also presented. (**b**) *Is[vit-2::GFP]* was stained with Nile Red after the fixation with paraformaldehyde. GFP (green), Nile Red (magenta), the merged image and the corresponding DIC image are presented. Bars, 5 μm.

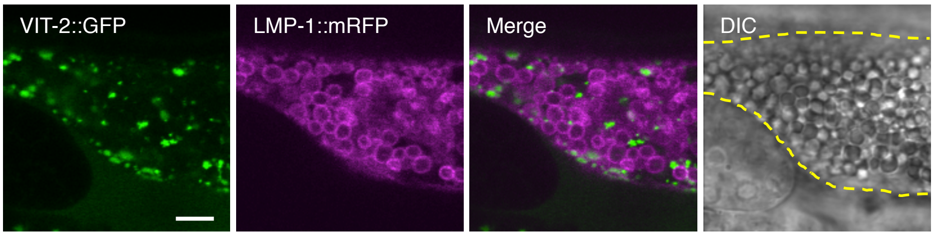

Figure S5 LMP-1::mRFP did not localize to VIT-2::GFP-positive granules. Fluorescence in the intestinal cells of *Is[vit-2::GFP];Is[ges-1p::lmp-1::mRFP]* was observed under confocal microscope. VIT-2::GFP (green), LMP-1::mRFP (magenta), the merged image, and the corresponding DIC image are presented. The intestine is indicated by yellow dashed lines. Bar, 5 μm.

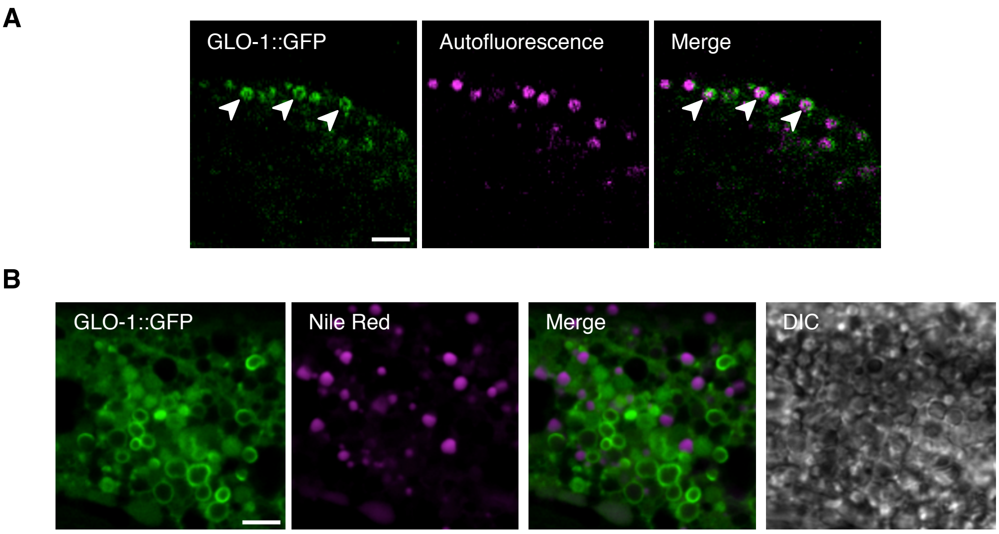

Figure S6 GLO-1::GFP-positive granules are autofluorescent but not Nile Red-positive. (**a**) Fluorescence observed in the int1 cell of *Is[ges-1p::glo-1::GFP]* under confocal microscope was unmixed to GFP (green) and autofluorescence (magenta). The merged image is also presented. Arrowheads indicate intestinal granules where GLO-1::GFP localize on the membrane. (**b**) *Is[ges-1p::glo-1::GFP]* was stained with Nile Red after the fixation with paraformaldehyde. GFP (green), Nile Red (magenta), the merged image and the corresponding DIC image are presented. Bars, 5 μm.

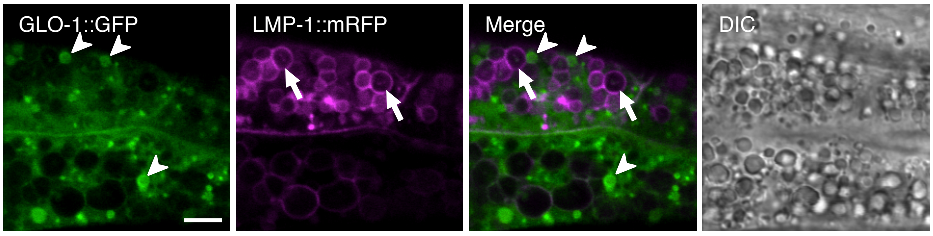

Figure S7 LMP-1::mRFP did not localize to GLO-1::GFP-positive granules. Fluorescence in the intestinal cells of *Is[ges-1p::glo-1::GFP];Is[ges-1p::lmp-1::mRFP]* at the L4 stage was observed under confocal microscope. GLO-1::GFP (green), LMP-1::mRFP (magenta) the merged image, and the corresponding DIC image are presented. Arrowheads indicate the intestinal granules where GLO-1::GFP localize on the membrane. Arrows indicate the LMP-1::mCherry-edged granules. Bar, 5 μm.

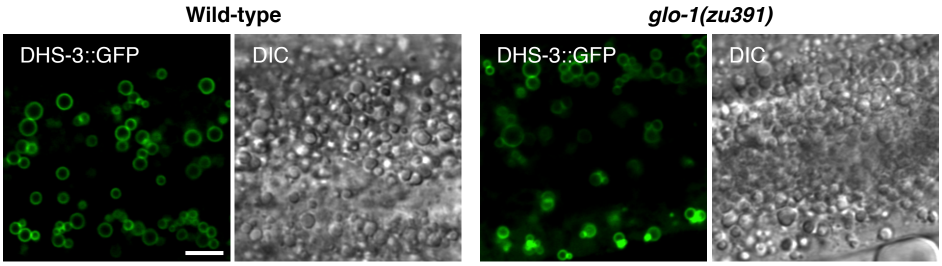

Figure S8 *glo-1* is not required for the biogenesis of DHS-3::GFP-positive lipid droplets. DHS-3::GFP (green) and the corresponding DIC images in *Is[dhs-3::GFP]* (left) and *Is[dhs-3::GFP];glo-1(zu391)* (right) are presented. Bar, 5 μm.

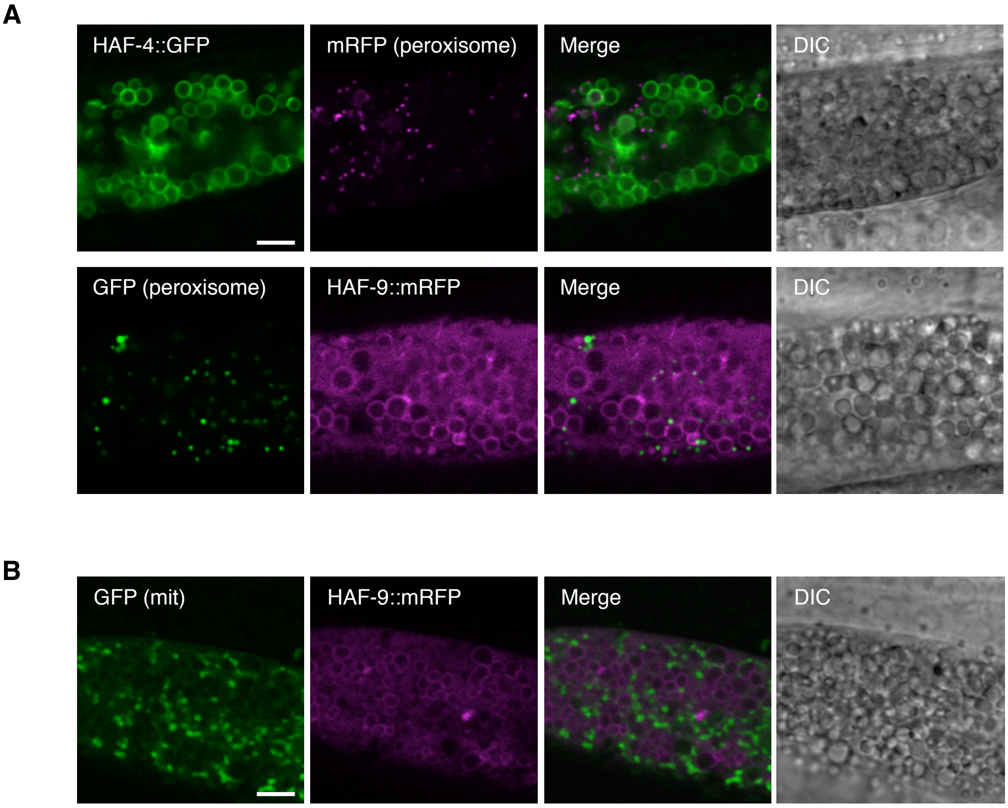

Figure S9 HAF-4 and HAF-9 did not localize to either the peroxisomes or the mitochondria.
(**a**) *Is[haf-4::GFP]* (top) and *Is[haf-9::mCherry]* (bottom) were crossed with the peroxisome marker mRFP-PTS1- and GFP-PTS1-expressing transgenic worms, respetively, and GFP (green) and mCherry (magenta) fluorescences were observed under confocal microscope. The merged images and the corresponding DIC images are also presented. GFP and mCherry signals were mutually exclusive in the merged images. (**b**) *Is[haf-9::mCherry]* were crossed with the mitochondrion marker GFP(mit)-expressing transgenic worms, and GFP (green) and mCherry (magenta) fluorescences were observed under confocal microscope. The merged image and the corresponding DIC image are also presented. GFP and mCherry signals were mutually exclusive in the merged images. Bars, 5 μm.

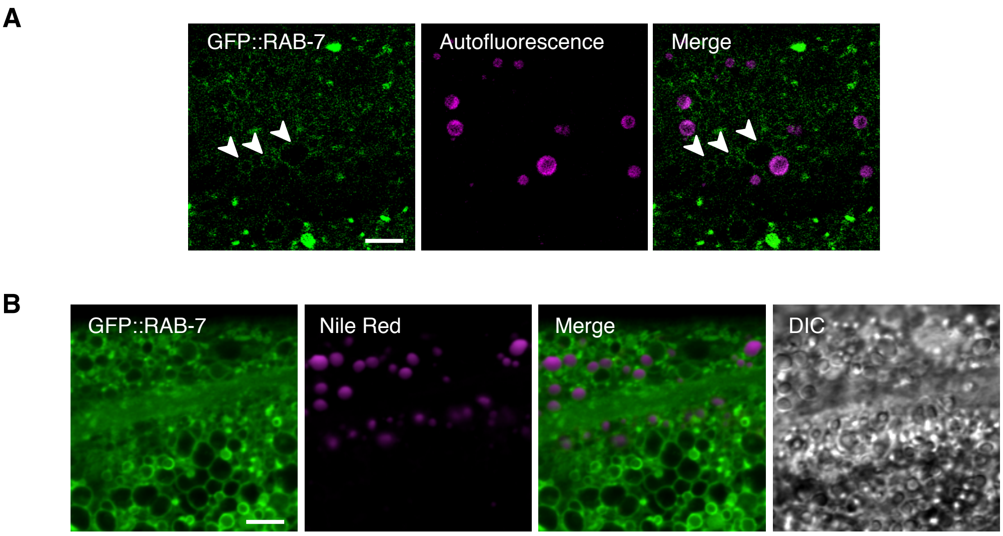

Figure S10 GFP::RAB-7 did not localize to either the autofluorescent granules or the Nile Red-positive lipid droplets.

(**a**) Fluorescence observed in the int2 cell of *Ex[GFP::rab-7]* under confocal microscope was unmixed to GFP (green) and autofluorescence (magenta). The merged image is also presented. Arrowheads indicate intestinal granules where GFP::RAB-7 localize on the membrane. (**b**) *Is[GFP::rab-7]* was stained with Nile Red after the fixation with paraformaldehyde. GFP (green), Nile Red (magenta), the merged image and the corresponding DIC image are presented. Bars, 5 μm.

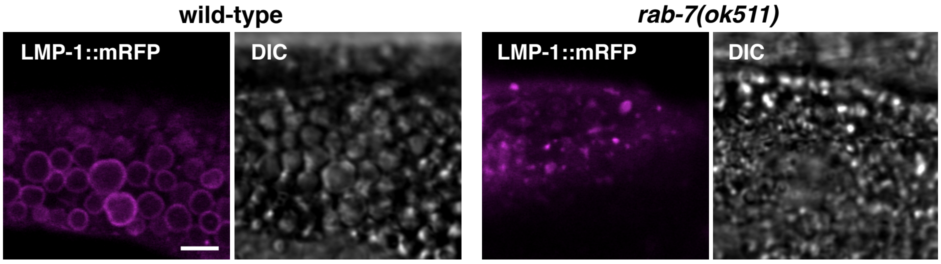

Figure S11 *rab-7* is required for the biogenesis of LMP-1::mRFP-positive granules. LMP-1::mRFP in the wild type (left) and *rab-7(ok511)* (right) at the L4 stage was observed under confocal microscope. The corresponding DIC images are also presented. LMP-1::mRFP-positive intestinal granules dramatically decreased in the *rab-7* mutants. Bar, 5 μm.

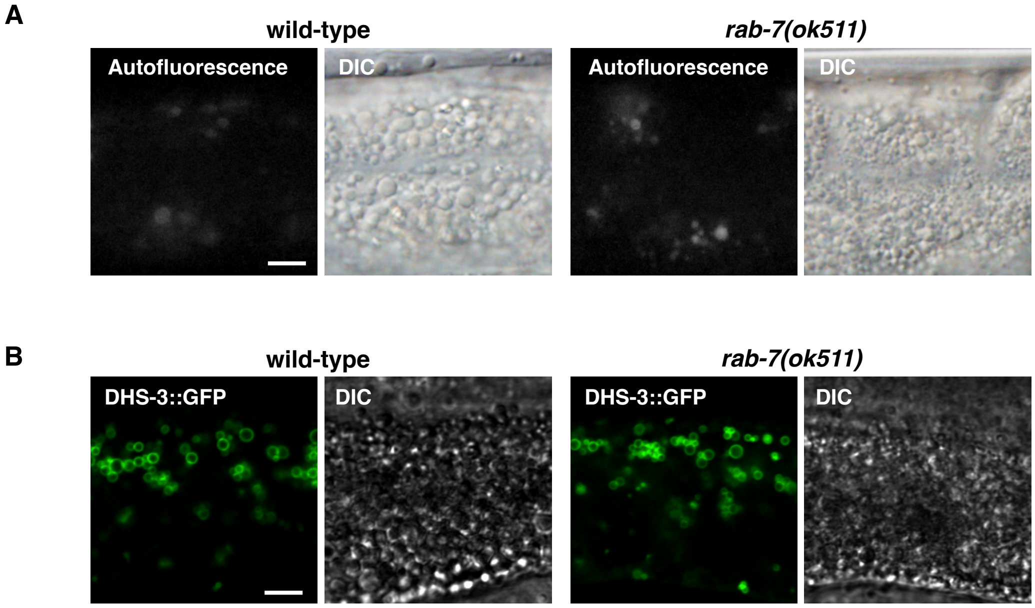

Figure S12 *rab-7* is not required for the biogenesis of autofluorescent granules and DHS-3::GFP-positive lipid droplets.

(**a**) Autofluorescence (shown in grayscale) in the wild type (left) and *rab-7(ok511)* (right) at the L4 stage was observed under epifluorescence microscope. The corresponding DIC images are also presented. Autofluorescent granules did not decrease in the *rab-7* mutants. (**b**) DHS-3::GFP-positive lipid droplets in the wild type (left) and *rab-7(ok511)* (right) at adulthood day 1 was observed under confocal microscope. The corresponding DIC images were also presented. DHS-3::GFP-positive granules did not decrease in the *rab-7* mutants. Bars, 5 μm.

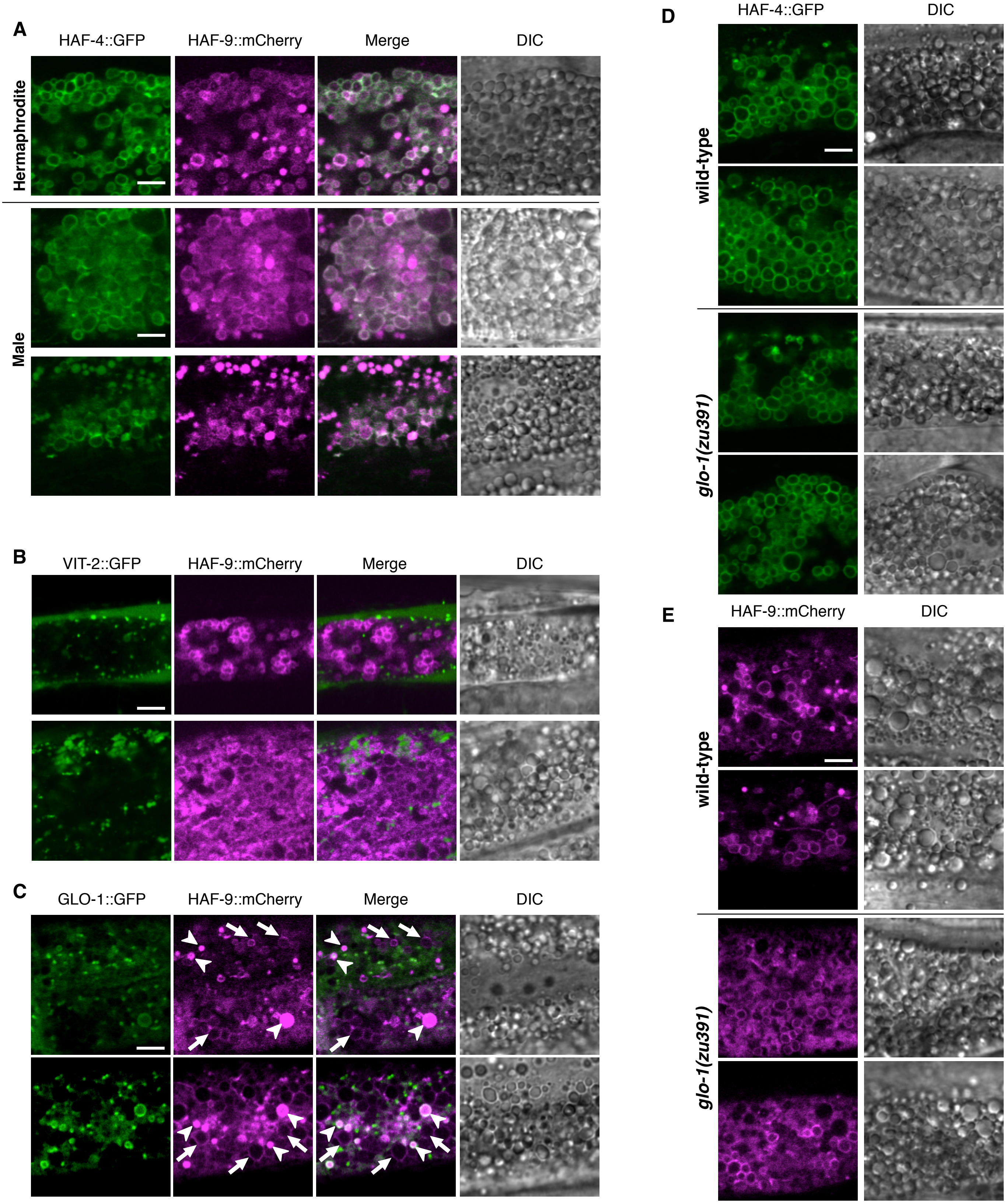

Figure S13 Other representative images.
Images from other animals than those in the main figures are presented. (**a**) Hermaphrodite and male animals of *Is[haf-4::GFP];Is[haf-9::mCherry]* (Figure 2a). (**b**) *Is[vit-2::GFP];Is[haf-9::mCehrry]* (Figure 2b). (**c**) *Is[ges-1p::glo-1::GFP];Is[haf-9::mCehrry]* (Figure 2b). Arrows indicate the mCherry-edged granules. Arrowheads indicate the accumulation of mCherry in the gut granules. (**d**) *Is[haf-4::GFP]* and *Is[haf-4::GFP];glo-1(zu391)* (Figure 3b). (**e**) *Is[haf-9::mCherry]* and *Is[haf-9::mCherry];glo-1(zu391)* (Figure 3b). Bars, 5 μm.

F

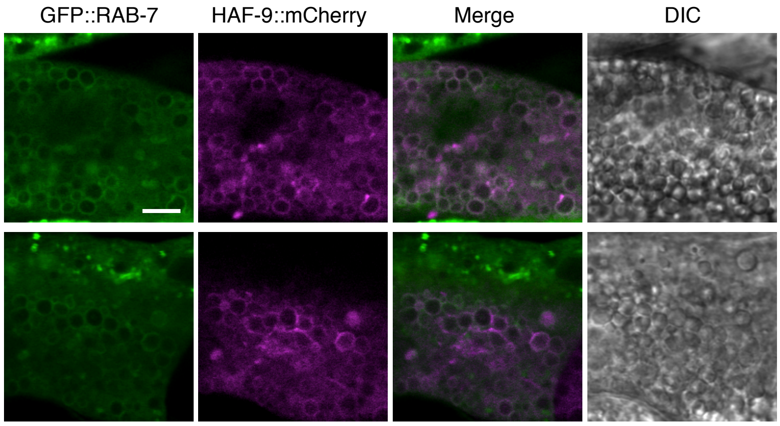

G

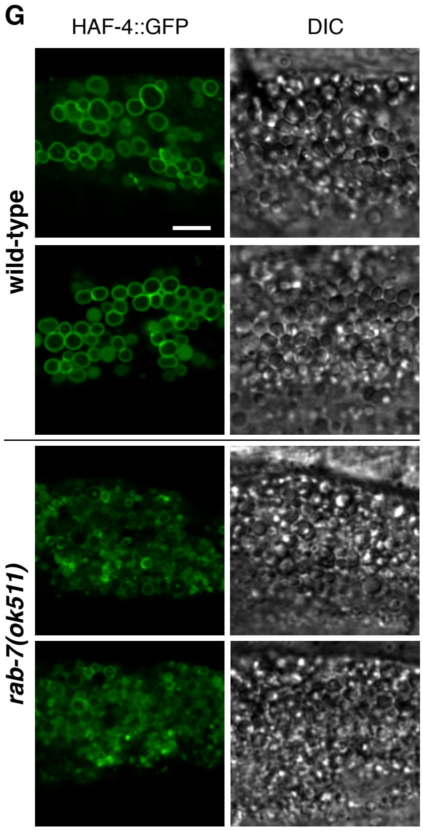

H

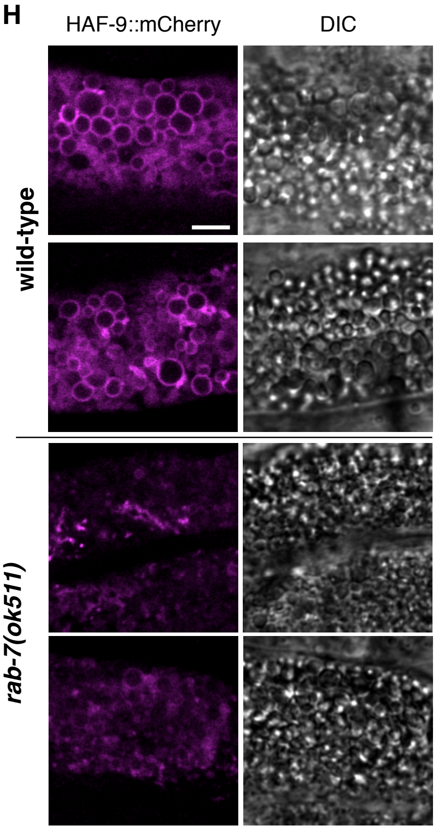

Figure S13 Other representative images (continued).
Images from two other animals than those in the main figures are presented. (**f**) *Is[haf-9::mCherry];Ex[GRP::rab-7]* (Figure 4a). (**g**) *Is[haf-4::GFP]* and *rab-7(ok511);Is[haf-4::GFP]* (Figure 4c). (**h**) *Is[haf-9::mCehrry]* and *rab-7(ok511);Is[haf-9::mCherry]* (Figure 4c). Bars, 5 μm.
